# The seroprevalence of *Mycobacterium avium subspecies paratuberculosis* in dairy cattle in Xinjiang, Northwest China

**DOI:** 10.1186/s13620-016-0079-0

**Published:** 2017-01-06

**Authors:** Xianxia Liu, Jianjun Li, Xueyun Yang, Dengfeng Wang, Jianmei Wang, Jianyong Wu

**Affiliations:** 1 College of Animal Science and Technology, Shihezi University, The North 4th Road, Shihezi, People’s Republic of China; 2 Veterinary Research Institute, Xinjiang Academy of Animal Science, NO. 726 Dongrong Street, Urumqi, People’s Republic of China

**Keywords:** *Mycobacterium avium subspecies paratuberculosis*, Seroprevalence, Dairy cattle, Farming mode

## Abstract

**Background:**

*Mycobacterium avium subspecies paratuberculosis* (MAP) causes chronic, wasting, and progressive enteritis in cattle, bringing significant economic losses in livestock industries. MAP has spread worldwide mainly due to movement of animals. The objective of this study was to determine the MAP seroprevalence in cattle in the Xinjiang Uygur Autonomous Region, Northwest China, and evaluate the difference between intensive farming herds (cattle number in a herd is more than 200, and the cattle cannot have access to pasture) and free-range herds (the cattle are bred by individual households, a herd is defined as the cattle are bred in a village or town in this study).

**Results:**

A total of 3157 serum specimens were collected from 42 herds in nine different regions. This included 1481 specimens from 18 intensive farming herds in four regions and 1676 specimens from 24 free-range herds in six regions. Antibody against MAP was tested with commercial ELISA test kits. The results showed that the overall apparent prevalence was 4.8% (95% CI, 4.1 to 5.6%) at animal level, and 50.0% (21/42) at herd level. The apparent prevalence in intensive farming herds and free-range herds were 9.5% (141/1481) and 0.7% (11/1676) at the animal-level, 88.9%(16/18) and 20.8% (5/24) at herd level, respectively, with a significant statistical difference between these two farming modes (*p* < 0.01). Cattle in intensive farming herds had a relatively higher risk to be infected with MAP than those in free-range herds (RR = 14.4).

**Conclusion:**

This study demonstrates that apparent prevalence of MAP infection in dairy cattle differs with farming modes at the animal level and herd level, and farming density could be an important risk factor associated with the presence of MAP infected cattle. This study provides important epidemiological data for bovine MAP control in Xinjiang, Northwest China.

## Background

*Mycobacterium avium subspecies paratuberculosis* (MAP) is the etiological agent of paratuberculosis or Johne’s disease, which causes severe chronic intestinal inflammatory disease in cattle and other ruminants [1]. Paratuberculosis is characterized by thickening of the intestinal wall, chronic intractable diarrhea, and progressive weight loss after infection [2]. Cattle are the most susceptible animals, particularly immature calves or heifers less than 1 - year of age. Animals are mainly infected via the faecal-oral route, through ingestion of faecal-contaminated composite feeds, water, and colostrum [3]. However, detection of MAP by antibody or pathogen determination is only effective after the animal has been infected at least 2 years, and typical clinical manifestations may require even more time [4]. Because of this inability to detect MAP at early stages, infected cattle remain in the herd and continue to shed MAP into the environment, causing more infections. Consequently, cattle paratuberculosis has resulted in great economic losses for the cattle industry, including a mean reduction of 5.9% in milk yield, or 1.9 kg/day in dairy cattle, and losses due to the animals losing weight and early death due to a lack of available treatment [5, 6].

Paratuberculosis is of increasing concern in many countries, such as Japan, Sweden, and Norway, which have implemented national strategies to control the disease in the past two decades [7–10]. Epidemiological investigations of paratuberculosis showed more than 50% of dairy cattle herds in Europe and USA were positive for the MAP antibody [11, 12]. Animal-level apparent prevalence has been reported with an average of 5–10% among cattle in USA, 1.2% in Belgium, and 8% in Denmark [11]. And in China’s neighbouring country Korea, the apparent prevalence was reported to be 3.3% at the animal level and 13.8% at the herd level [13].

In China, there has been some recent works to determine the serological patterns of MAP. An epidemiological investigation of paratuberculosis was performed in Shandong province, east China, the reports found that 11.7% (121/1038) of dairy cattle and 57.9% (11/19) of dairy cattle herds tested positive for the MAP antibody [14]. Similarly, 11.8% (433/3674) of cattle in Heilongjiang, Jilin, Liaoning, and inner Mongolia, in northeast China [15], and 17.6% in sika deer in Jilin province, in northeast China were serologically positive for MAP [16]. Although China is one of the largest cattle-producing countries, there is incomplete epidemiological data for cattle paratuberculosis, especially the data about different farming modes.

In this study, a survey was performed in Xinjiang Uygur Autonomous Region. In this region, there are 3.6 million cattle, including 3.0 million free-range cattle and 0.6 million intensively housed cattle. The aim of the survey was to determine the animal-level and herd-level MAP apparent prevalence in both farming modes.

## Methods

**Fig. 1:**
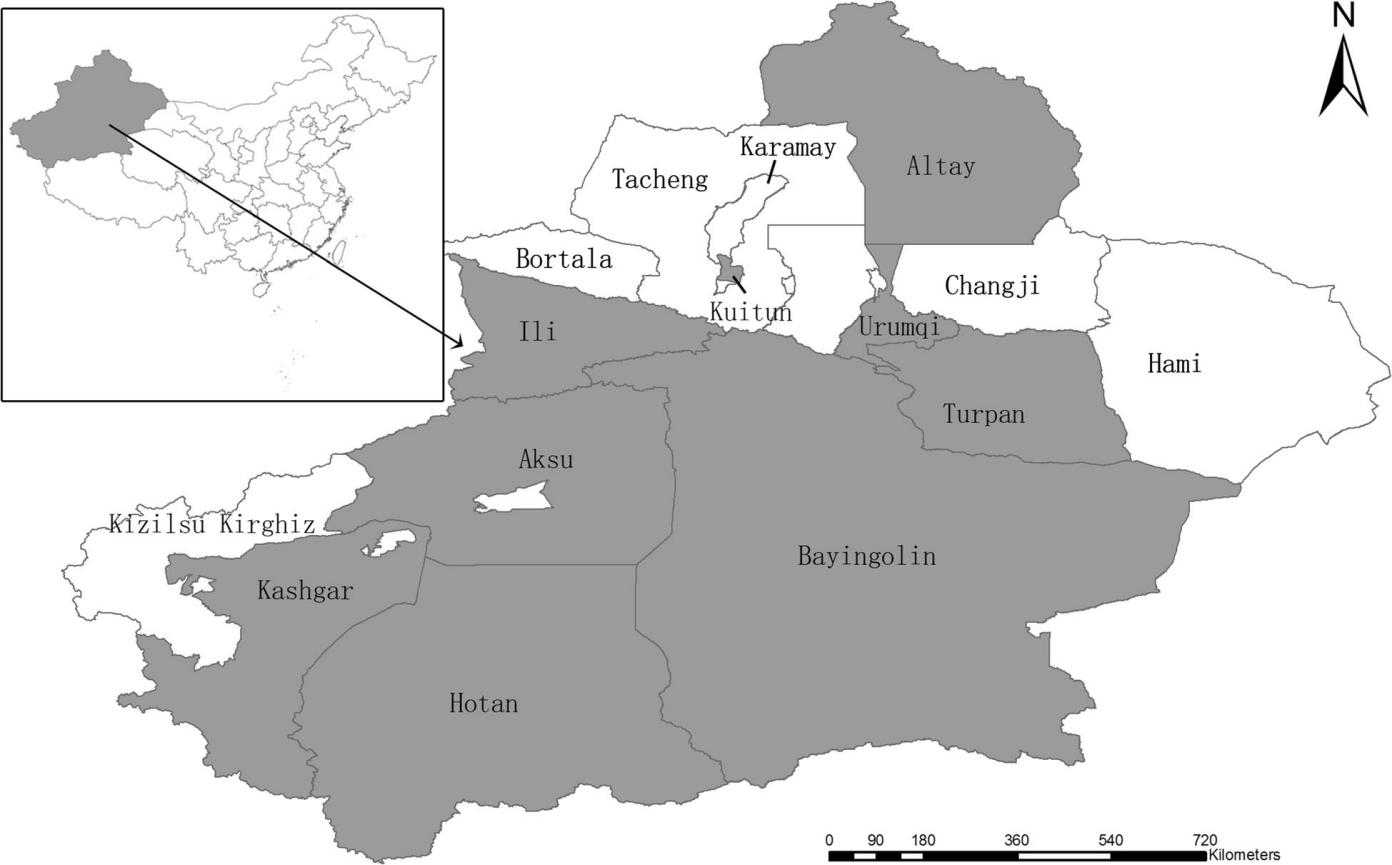
Geographic distribution of specimens collected from Xinjiang, northwest China. Specimens were collected from those regions indicated in grey

The sample size calculated method was performed as used by Kim, et al for a sample size of 643 [17]. In this study we collected serum specimens from 3157 lactating cows to increase data reliability. The specimens were sampled from nine regions of Xinjiang, which were located in the major dairy farming belt and farming and pastoral areas, including Urumqi (584 specimens, 4 herds), Kuitun (306 specimens, 7 herds), Ili (279 specimens, 4 herds), Altay (279 specimens, 2 herds), Bayingolin (372 specimens, 5 herds), Khotan (471 specimens, 6 herds), Turpan (303 specimens, 5 herds), Aksu (528 specimens, 8 herds), and Kashgar (35 specimens, 1 herds), between May and July 2015. The specimens from intensive farming herds were collected in Urumqi, Kuitun, Ili, and Aksu, where cattle are bred in an intensive farming mode. The specimens from free-range herds were collected in Altay, Bayingolin, Khotan, Turpan, Aksu, and Kashgar, where cattle were allowed to graze and roam freely (Fig. 1). All cattle appeared clinically healthy at the time of sampling, and all cows were at least 2 years old. Cattle blood specimens (5 ml) were sampled by puncturing the tail vein using sterile tubes. Specimens were stored at 4 °C before being centrifuged at 1000 × g for 15 min. Sera were separated and stored at −20 °C until further testing. Serum specimens were analyzed with the commercial ELISA kits (*Mycobacterium paratuberculosis* antibody test kit, IDEXX Laboratories) according to the manufacturer’s instructions.

Descriptive statistics were used to determine the apparent prevalence of MAP at the animal and herd level, and for animals in the different farming modes. The results were analyzed using the SPSS 22.0 software package, the chi-squared test was used to analyze the MAP-positive cattle between intensive farming herds and free-range herds, and then to compare the differences in cattle in the two farming modes based on the percentage of positive cattle in those two groups. The rate ratio (RR) and 95% confidence interval (CI) were also calculated. A *p* value less than 0.05 was considered statistically significant.

## Results

A total of 152 specimens out of the 3157 sera tested (4.8%) (95% CI, 4.1 to 5.6%) were positive for the MAP antibody. 141 specimens (9.5%) (95% CI, 8.0 to 11.0%) were positive for MAP antibody of the 1481 specimens collected from the intensive farming herds in Kuitun, Aksu, Urumqi and Ili, where the positive rates were 4.9% (95% CI, 2.5 to 7.3%), 21.2% (95% CI, 16.6 to 25.7%), 7.9% (95% CI, 5.7 to 10.1%) and 5.0% (95% CI, 2.5 to 7.6%), respectively. Regarding the 1676 specimens from the free-range herds sampled from six regions, 11 were seropositive, accounting for only 0 · 7% (95% CI, 0.3 to 1.0%). The positive specimens were found only in three regions, Khotan (0.4%, 95% CI, 0 to 1.0%), Bayingolin (0.3%, 95% CI, 0 to 0.8%), and Altay (2.9%, 95% CI, 0.9 to 4.8%). No positive specimens were detected in Kashgar, Turpan, or Aksu (Tables 1 and 2).

**Table 1:** Apparent prevalence of cattle MAP in different regions of Xinjiang, China

Region	Herd level		Animal level	
	Numbers of herds	Positive number	Prevalence (%) (positive no/No tested)	95% CI (%)
Kuitun^a^	7	5	4.9 (15/306)	2.5 to 7.3
Aksu^a^	3	3	21.2 (66/312)	16.6 to 25.7
Urumqi^a^	4	4	7.9 (46/584)	5.7 to 10.1
Ili^a^	4	4	5.0 (14/279)	2.5 to 7.6
Kashgar^b^	1	0	0.0 (0/35)	0
Turpan^b^	5	0	0.0 (0/303)	0
Khotan^b^	6	2	0.4 (2/471)	0 to 1.0
Aksu^b^	5	0	0.0 (0/216)	0
Bayingolin^b^	5	1	0.3 (1/372)	0 to 0.8
Altay^b^	2	2	2.9 (8/279)	0.9 to 4.8
Total	42	21	4.8(152/3,157)	4.1 to 5.6

^a^ Serum specimens were collected from intensive farming herds

^b^ Serum specimens were collected from free-range herds

**Table 2:** Apparent prevalence of MAP in different farming modes in Xinjiang, China

	Herd level		Animal level	
Farming mode	Prevalence (%) (positive no/No tested)	95% CI (%)	Prevalence (%) (positive no/No tested)	95% CI (%)
Intensive farming	88.9 (16/18)	65 to 99	9.5 (141/1,481)	8.0 to 11.0
Free-range	20.8 (5/24)	7 to 42	0.7 (11/1,676)	0.3 to1.0
Total	50.0 (21/42)	34 to 66	4.8 (152/3,157)	4.1 to 5.5

Twenty-one out of 42 herds (50.0%) were found serologically positive for MAP antibody. For intensive farming herds, 88.9% (16/18) were positive including 71.4% from Kuitun (5/7) and 100% from Aksu (3/3), Urumqi (4/4), and Ili (4/4). 20.8% (5/24) of free-range herds from Kashgar (0/1), Turpan (0/5), Khotan (2/6), Aksu (0/5), Bayingolin (1/5) and Altay (2/2) were positive for MAP antibody (Tables 1 and 2).

When the difference between the two farming modes was compared, apparent prevalence among intensive farming herds was significantly higher than that in the free-range herds at the animal level (chi-squared = 134 · 8, *p* < 0 · 01), and also at the herd level (chi-squared = 19.1, *p* < 0 · 01). The cattle farming in the intensive farming mode had a relatively higher risk than that in the free-range mode (RR = 14.4).

## Discussion

The current study was the first serological survey of MAP infection in both intensive farming herds and free-range herds in the Xinjiang Uygur autonomous region, which is the largest provincial level district with 1.6 million square kilometres area in China. The results indicated that 4.8% (95% CI, 4.1 to 5.6%) of cattle and 50.0% (21/42) of herds were detected as serologically positive for MAP antibody, which was lower than in previous studies from other provinces in China. This is mainly due to this study containing specimens from both intensively farmed and free range herds.[14–16]. Previous studies in other provinces of China did not include data from free-range herds, which account for large proportion of animals in the current cattle breeding industry. So, this study could manifest a reliable epidemic status of MAP infection in Xinjiang.

For a sampling design, it would be better to select animals randomly from a master list, however, this study was not put into practice completely due to lack of practicability. Instead, cattle and herds were selected through the local veterinary authorities, who are responsible for the annual spring blood testing (test the infectious diseases such as Brucellosis, Tuberculosis, and others, in spring every year) in China. The degree of convenience sampling differs from regions and herds (35 animals per herds in Kashgar to 146 in Urumqi). Convenience sampling may bias the results of cross-sectional studies. However, the specimens in this study covered more herds and regions, and also included different farming modes to reduce selection bias.

Interestingly in this survey, the MAP prevalence differed from breeding modes, 9.5% (95% CI, 8.0–11.0%) of cattle were detected as positive in intensive farming herds, while only 0.7% (95% CI, 0.3–1.0%) were positive in free-range herds, which was showed statistical difference in these two farming modes (*p* < 0 · 01). Furthermore, we confirmed the effect of animal level on the prevalence of MAP infection, and the level in intensive farming herds was statistically significantly higher than those of free-range herds. The RR value was 14.4, implied that the risk in intensive farming herds was even higher than that in free-range herds. The possible reasons of this association might be as follows: Firstly, it was different management practices, such as feeding pooled colostrum, breeding calves together in large herds and faecal contamination of composite feed and water, all of which could result in an increasing incidence of within-herd transmission of MAP via faecal-oral transmission. Secondly, incidence of paratuberculosis varied from the herds size. MAP was identified in 95% of large dairy herds (>500 cows), compared with only 63.2% of small herds (<100 cows) [18], indicated that farming scale or breeding density was an important risk factor of MAP infection. Thirdly, ﻿a large number of cattle were imported from foreign countries, such as Australia, New Zealand and Canada, in the first decade of the 21st century to reconstruct high producing dairy cow population in Xinjiang. Paratuberculosis has been reported in all of these countries although adopting appropriate entry-exit inspection and quarantine at the time of importation might not have been effective due to the potential latent nature of MAP infection.

Additionally, the apparent prevalence of cattle in Xinjiang at both animal and herd levels were more serious than that of neighbouring countries, such as Korea. The reasons may be attributed to practice management in farms as well as a lack of a MAP control plan in China. Admittedly, the most critical problem was not established national control strategy to conduct MAP prevention. In Japan and Sweden, national control strategies have proven to be effective in preventing MAP spreading, keeping it under an acceptable incidence or eradicating it [7–10]. Recently, MAP has received an increasingly wide interest because of a rapidly growing body of scientific evidence which suggests that human infection with this microorganism may be causing some, and possibly all, cases of Crohn’s disease [19, 20].

This paper also has some limitations. 1) This was a cross-sectional study, the incidence of cattle paratuberculosis at the animal level could not be inferred because of the imperfection of the cross-sectional study itself. 2) This study did not find the risk factors of high prevalence of cattle MAP infections in Xinjiang, just hypothesize the possible reasons. In future research, prospective studies, such as cohort studies or case control studies would be able to find clues of high prevalence of cattle MAP further in Xinjiang, northwest China.

## Conclusion

This study reported the apparent prevalence of MAP infection in dairy cattle in Xinjiang, northwest China, and found that farming density was an important risk factor associated with the presence of MAP infected cattle. National and provincial quarantine and eradication measures should be strengthened in intensive farming herds to reduce MAP infection and limit its spread. Future studies are warranted to determine the molecular epidemiology and genomic characteristics of MAP in China.

## Abbreviations

CI: Confidence interval
ELISA: Enzyme-linked immunosorbent assay
MAP: *Mycobacterium avium subspecies paratuberculosis*
RR: Rate ratio
